# Gaps in Heat-Related Knowledge, Practices and Adaptation Strategies Among Coaches in German Outdoor Sports

**DOI:** 10.3389/ijph.2024.1607928

**Published:** 2024-12-04

**Authors:** Sophie Leer, Zoe A. Parsons, Sven Schneider

**Affiliations:** Division of Public Health, Social and Preventive Medicine, Center for Preventive Medicine Baden-Württemberg, Medical Faculty Mannheim, University of Heidelberg, Mannheim, Germany

**Keywords:** outdoor sports, coaches, health risks, heat stress disorders, heat illness

## Abstract

**Objectives:**

Climate change is increasing the risk of heat-related illness in outdoor sports. Coaches have a responsibility to protect the athletes in their care. In this study, the knowledge and practice of German coaches in heat prevention were evaluated nationwide.

**Methods:**

Coaches (n = 1,200) from the ten largest outdoor sports in Germany were asked about their knowledge using the knowledge of heat-related illness symptoms index (KOSI, range [0–14]). Prevention measures currently implemented by coaches were analyzed in terms of relative compliance with specified recommendations (heat prevention score (HPS), range [0–100]).

**Results:**

The KOSI averaged 10.31 ± 1.81 and pointed to clear knowledge deficits: the lowest score values were shown by coaches in skiing (9.85 ± 1.80), soccer (10.07 ± 2.33) and golf (10.09 ± 1.75; pANOVA = 0.015). Heat protection in training was also deficient: The HPS showed a mean value of 62.41 ± 14.89. The greatest deficits existed in tennis (57.71 ± 14.29), mountain sports (58.17 ± 13.08) and soccer (58.70 ± 13.86; pANOVA < 0.001). No correlation between theoretical knowledge and practical prevention was found.

**Conclusion:**

In Germany, coaches are insufficiently prepared for the health hazards of heat. Promoting onsite educational programs seems essential to ensure safer sports environments.

## Introduction

The significant increase in heatwaves is considered to be the most important health consequence of climate change. According to the World Health Organization (WHO), in addition to children, pregnant women and multimorbid seniors, athletes are particularly at risk in this context [1]. The reason is that in outdoor sports, unlike other risk groups, it is almost or even completely impossible to seek out air-conditioned rooms or avoid physical activity during a heatwave.

Doing sport in a hot environment is much more strenuous for the body than in cooler conditions [2]. Prolonged exposure to heat can lead to heat-related illnesses [3]. Heat-related illnesses include heat syncope, heat cramps, heat exhaustion and exertional heat stroke (EHS) [4]. Regarding EHS, a recent narrative review critizises that to date there is neither a register to record valid incidences nor an internationally standardized definition [5]. Nevertheless, studies on long-distance road races found EHS incidences between 1.6 und 2.1 per 1,000 finishers (without mortality; 5). At least when ambient temperatures are above average (median start-time temperature 24°C/75°F), exertional heat strokes are even more common than non-heat-related cardiac events in endurance competitions [6]. Last but not least, exertional heat stroke is one of the leading causes of death among athletes [7].

Sports organizations, clubs and coaches have a responsibility to protect the athletes in their care from these risks. This applies first to competitive sports. Competitive athletes are particularly exposed to heat risks due to the long seasons, high training volumes and high competition density. However, the risks of heat-related illnesses should not be underestimated in amateur sports either due to recreational athletes generally having a less favorable fitness and acclimatization status, meaning that the risk of developing a heat-related illness ceteris paribus may even be higher compared to a competitive athlete [8]. Even with a lower training intensity, a less favorable movement economy (i.e., milliliters of oxygen per kilogram of total body mass per kilometer run) can lead to a comparatively higher heat production in this case [9]. Apart from this, the population of recreational athletes is significantly larger in number compared to competitive athletes and therefore the cumulative risk for an entire population is higher.

Following recent suggestions [10, 11], the present study examines knowledge of heat risk prevention, prevention practices, and opportunities for prevention among coaches working in German outdoor sports, as well as differences in knowledge and practices between various types of sports.

## Methods

### Study Design and Setting

As part of the “C^3^O study” (*C*limate *C*hange, *C*oaches and *O*utdoor Sports - *Study*) presented here for the first time, coaches were asked about prevention knowledge, practice and options for action in relation to heat stress in sport as part of a cross-sectional study. The survey was conducted nationwide and the ten largest outdoor sports associations in Germany were included. We followed the definition of Dee et al. [12], according to which it is an outdoor sport if the competitions take place outdoors. According to the official membership statistics of the umbrella organization, the German Olympic Sports Confederation (DOSB), these were the German Football Association (DFB), the German Tennis Association (DTB), the German Alpine Association (DAV), the German Athletics Association (DLV), the German Equestrian Federation (FN), the German Golf Association (DGV), the German Life Saving Association (DLRG), the German Ski Association (DSV), the German Sailing Association (DSV) and the German Cyclists’ Federation (BDR). With a total membership of 14 million people, 16% of the German population are organized in these associations (as of 01/2023) [13, 14]. Examples of specific heat exposure include the German Athletics Association (DLV), which represents all athletics disciplines, the German Life Saving Association (DLRG), which is responsible for swimming training throughout Germany, and the German Sailing Association (DSV), where infrared and UV exposure is amplified by water reflection.

To reach as many coaches of these sports as possible, the national and local sports associations were contacted, numerous study calls were published online and in the printed association notices and further participants were recruited via e-mail and telephone directories of the sports associations. All participants were informed in advance via an online link about the study content and procedure. Informed consent was obtained from all individual participants included in the study. The survey took place as an asynchronous written online survey using computer aided web interview (CAWI) with the help of a standardized questionnaire using the Lime-Survey software package from May 2022 to June 2023. Before the start of the survey phase, the online questionnaire was subjected to an expert review (n = 4) and a standard pre-test (n = 8 coaches excluded from final sample). Suggestions and ideas from the expert reviews and the pretest were then incorporated into the final questionnaire (e.g., the suggestion to ask the coaches from skiing the knowledge questions, but not the questions on prevention behavior, which is irrelevant for them in practice). All procedures performed in studies involving human participants were in accordance with the ethical standards of the institutional committee (ethics committee of the Medical Faculty Mannheim of the University of Heidelberg) and with the 1964 Helsinki declaration and its later amendments or comparable ethical standards: The STROBE Statement [15] on standardized reporting of cross-sectional studies were met. An unreservedly positive vote of the responsible ethics committee of the Medical Faculty Mannheim of the University of Heidelberg was available in advance (AZ 2021-653, 16 November 2021). The study was preregistered in the German Clinical Trials Registry on 18 January 2022 (registration number DRKS00027815; https://drks.de/search/de/trial/DRKS00027815).

### Participants

In addition to being of legal age (≥18 years) and a German resident, the central inclusion criterion was a coaching activity in competitive or popular sports in one of the above-mentioned outdoor sports. The study participants were recruited by means of quota sampling, a systematic, non-probabilistic sampling procedure, according to type of sport and federal state. The minimum sample size of n = 600 originally envisaged in the study protocol was far exceeded by the actual participation (n = 1,200). Using a standard weighting procedure (so-called standard redressment on number of respondents), each of the above-mentioned outdoor sports was represented with 120 respondents (disproportionate quotas by type of sport) and the sample was representative nationwide in terms of distribution across the 16 federal states (proportional quota by federal state; for details see Supplementary 1, [16]).

### Variables and Measurement

In addition to descriptive information (e.g., to check the inclusion criteria such as age, license level, type of sport, federal state of the club location), the research questions were operationalized as follows in the questionnaire:

Expected impact of climate change: all coaches were first asked in general terms how likely they thought climate change would have an impact on the health of outdoor athletes within the next 10 years (in %).

Prevention knowledge (Knowledge of heat-related illness symptoms index - KOSI): Whether trainers know the typical symptoms of heat-related illnesses was recorded using a battery of items. This was developed by Smith et al. [17] based on recommendations of the National Institute for Occupational Safety and Health [18] and was kindly made available to us by the authors. This list of symptoms largely coincides with relevant reviews and expert reports [4, 19]. The question “Which of the following happens to a person who is sick from heat exposure?”, ten applicable symptoms (headache, dizziness, heavy sweating, irritability, nausea and vomiting, slurred speech, seizures, confusion, fainting, very high body temperature) and four non-applicable symptoms (blisters on skin, pain while urinating, rash on hands, lower back pain) were asked. A sum index with the value range [0–14] was calculated from all correct answers. The operationalization of the KOSI together with the original questionnaire and the validation results can be found in detail as a supplement by Smith et al. [17].

Prevention practice and options for action (heat prevention score): The survey of prevention measures currently implemented by coaches when training in hot weather was based on the recommendations “Exercise in the Heat” [20] published by the German Society for Sports Medicine and Prevention (DGSP). Essentially, these ten DGSP recommendations concretize the guidelines of the German Society for General Medicine [21] and the WHO guidelines [22] for the sports context. The trainers were asked to imagine a “cloudless, sunny summer day with an outside temperature of over 30°C” (=86°F) and to indicate on a 100-point Likert scale with the poles 0 = “never” vs. 100 = “always” how often the respective measure is currently implemented in a typical training session under these conditions (prevention practice) and how easy it is or would be to implement in principle (options for action). The ten individual measures were also summarized in two sum indices (heat prevention scores HPS; range [0–100]) according to the general calculation formula:

$$HPS=\left({\text{LV}}_{\text{RA}}+{\text{LV}}_{\text{RB}}+{\text{LV}}_{\text{RC}}+{\text{LV}}_{\text{RD}}+{\text{LV}}_{\text{RE}}+{\text{LV}}_{\text{RF}}+{\text{LV}}_{\text{RG}}+{\text{LV}}_{\text{RH}}+{\text{LV}}_{\text{RI}}+{\text{LV}}_{\text{RJ}}\right)/10$$

with:

LV = Likert scale value.

_R(A-J)_ = Recommendation A-J.

A = Movement of training to the morning or evening hours.

B = Reduction of exercise intensity and duration.

C = Drinking sufficient fluids before training.

D = Drinking sufficient fluids during training.

E = Drinking sufficient fluids after training.

F = Application of external cooling methods during and after training.

G = Wearing of suitable, light and airy clothing.

H = Wearing of headgear during training breaks.

I = Observation of athletes for heat symptoms during training.

J = Presence of medical personnel at organised competitions.

### Bias and Quality Control

The online survey method enabled plausibility checks to be carried out during entry, for example, by blocking unauthorized values and incompletely filled out questionnaires. Multiple participations from the same IP address were also not permitted. In addition, plausibility checks were carried out in the data set after the data base lock (30 June 2023). They did not reveal any systematic anomalies. The average processing time was 24 min and over 95% of the respondents took between ten and 60 min to complete the questionnaire. The original questionnaire can be obtained from the corresponding author.

### Statistical Analysis

The C^3^O study was analyzed using classical descriptive and inferential methods. The metric scores were approximately normally distributed. Arithmetic mean and median were less than 0.2 standard deviations apart. Group differences were analyzed using unpaired t-tests and one-way ANOVA, bivariate correlations of the scores using Pearson’s *r* ^2^. The analyses were performed using the program IBM SPSS Statistics 29.0.0 (IBM Corp., Armonk, United States). All tests were exploratory and two-sided with a significance threshold of *p* ≤ 0.05.

## Results

The sample of coaches had an average age of 44 (±14, range [18–81]) years and was 68% male. On average, the participants could look back on 15 (±11) years of experience as trainers. The training group comprised an average of 11 (±8) people and consisted of children in 26% of the cases, adolescents in 40% and adults in 34%. Gender was missing in two cases and age was missing in three cases. All other variables had no missing values.

**FIGURE 1:**
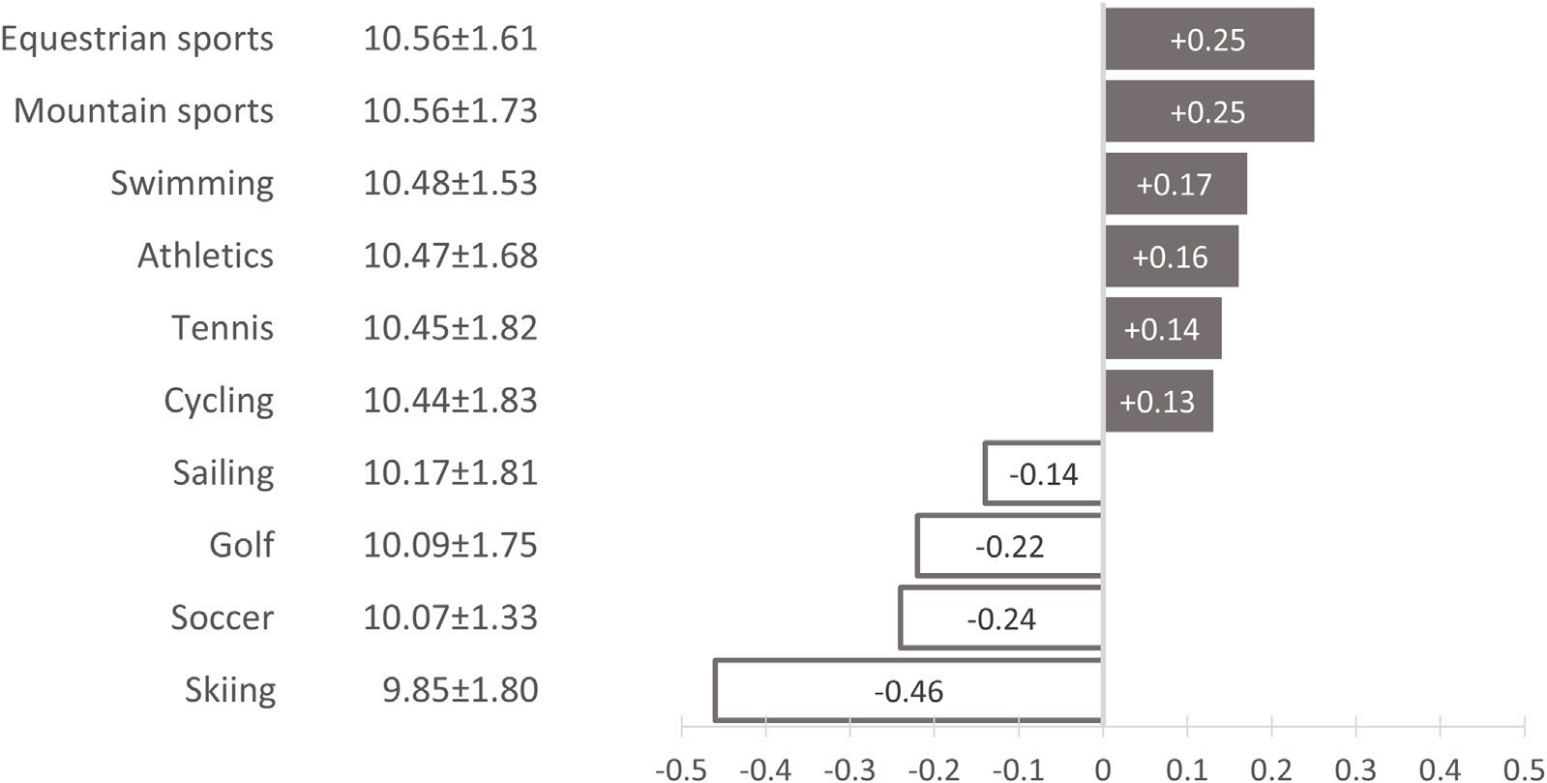
Heat-related health knowledge in German outdoor sports by type of sport. Absolute deviation from the arithmetic mean of the knowledge of heat-related illness symptoms index (KOSI) by type of sport (arithmetic mean: 10.31 ± 1.81; ANOVA: F-value: 2.29, df = 9, *p* = 0.015); Climate Change, Coaches and Outdoor Sports – Study (C^3^O study), Germany, 2022–2023. Grey = Positive deviation from the mean value. White = Negative deviation from the mean value.

Overall, the trainers estimated the probability of climate change having an impact on the health of outdoor athletes at an average of 62% (±29.29). The KOSI scores, which measures knowledge of typical heat-related illness symptoms, averaged 10.31 (±1.81, range [3–14]). The least known was that symptoms such as heavy sweating, slurred articulation, seizures and irritability can be symptoms of heat-related illness. Only 61.7%, 50.3%, 39.8% and 33.4% of all trainers were aware of this in relation to the individual symptoms. There were also significant differences in heat-related health knowledge between the sports (Figure 1, ANOVA: F-value: 2.29, df = 9, *p* = 0.015): The least favorable values were shown by coaches in skiing and soccer, the best values by coaches in mountain sports and equestrian sports.

**FIGURE 2:**
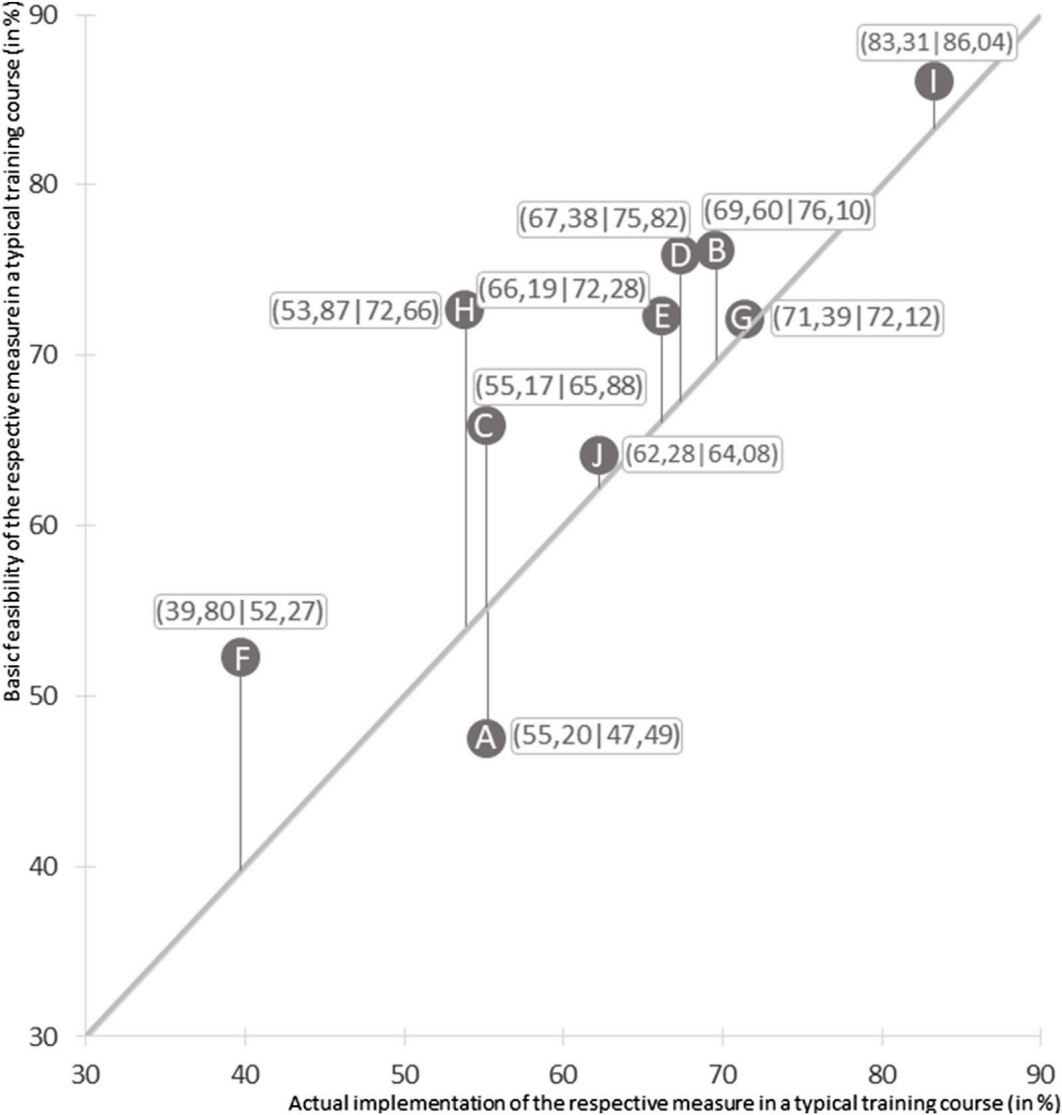
Implemented and possible heat protection measures in German outdoor sports. Measures taken by “the majority of the training group on a cloudless, sunny summer day with an outside temperature of over 30°C” (=86°F); n = 1,080 (without the coaches in skiing). Climate Change, Coaches and Outdoor Sports – Study (C^3^O study), Germany, 2022–2023. Prevention measures based on the recommendations of the German Society for Sports Medicine and Prevention: A = Movement of training to the morning or evening hours. B = Reduction of exercise intensity and duration. C = Drinking sufficient fluids before training. D = Drinking sufficient fluids during training. E = Drinking sufficient fluids after training. F = Application of external cooling methods during and after training. G = Wearing of suitable, light and airy clothing. H = Wearing of headgear during training breaks. I = Observation of athletes for heat symptoms during training. J = Presence of medical personnel at organised competitions.

The coaches stated that on a cloudless summer day with temperatures above 30°C, their athletes were most likely to pay attention to heat symptoms (83.31 ± 24.19), light, airy clothing (71.39 ± 28.10) and adapted training intensities (69.60 ± 28.19) (Figure 2). In contrast, the trainers stated that only around two-thirds of all training groups drank enough during training in accordance with the recommendations (67.38 ± 27.76). According to their reports, sufficient fluid intake before and after training was even less common (55.17 ± 30.20 and 66.19 ± 25.83). The additional question of how easy it would be to implement each individual measure during training provided an indication of the potential for improvement. Figure 2 shows the percentage value for the possible implementation of the respective measures using the *y*-axis coordinate. The vertical lines illustrate that, in addition to wearing headgear, the provision of external per- and post-cooling measures (such as cool packs, spray bottles, ice for rubbing and moist towels) and a better supply of fluids before training have the greatest potential for improvement (Figure 2).

**FIGURE 3:**
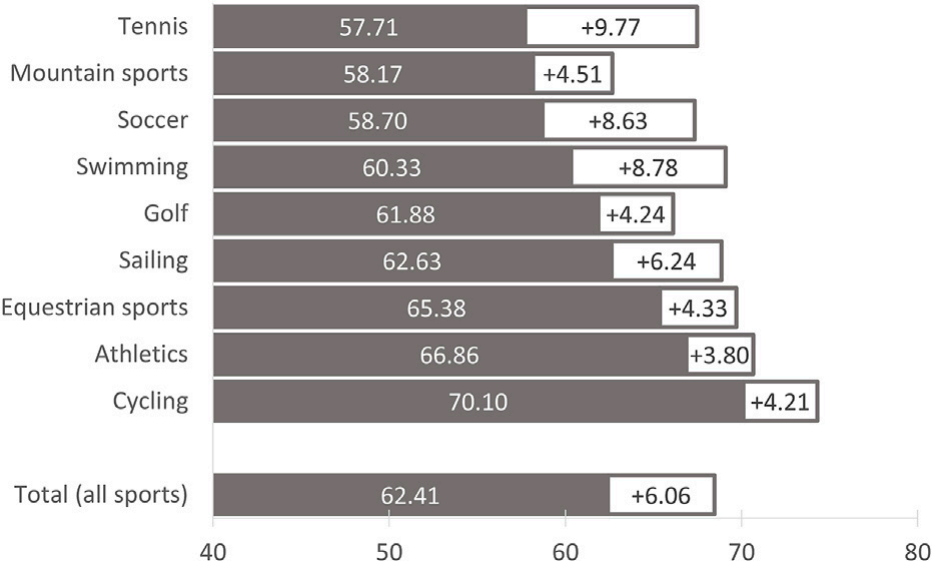
Implementation and potential for improvement in heat protection measures in German outdoor sports by type of sport. Climate Change, Coaches and Outdoor Sports – Study (C^3^O study), Germany, 2022–2023. n = 1,080 (without the coaches in skiing). Grey = Degree of implementation (%), sum index of the ten prevention measures based on the recommendations of the German Society for Sports Medicine and Prevention (for details see legend of Figure 2 and Ref. [20]). White = Potential for improvement (%), difference between the sum index of possible heat protection measures and the sum index of measures implemented.

The current prevention practice (ANOVA: F-value: 10.59, df = 8, *p* < 0.001) and the possible options for action (ANOVA: F-value: 5.34, df = 8, *p* < 0.001) differed significantly between the sports. The most striking deficits in the preventive measures applied can be seen in tennis, mountain sports, soccer and swimming (Figure 3). If the coaches are asked about implementation possibilities, the greatest potential for improvement is also evident in these sports - except for mountain sports (with index values of 67.48 ± 14.29, 62.68 ± 14.95, 67.33 ± 17.62, and 69.10 ± 16.43; for differences see Figure 3).

**FIGURE 4:**
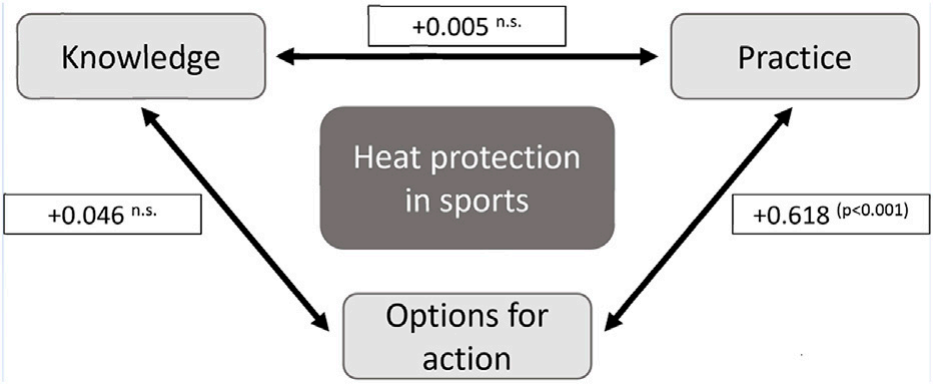
Correlation of prevention knowledge, practice and options for action of coaches in German outdoor sports. Pearson’s *r* ^2^, n = 1,080 (without the coaches in skiing). n.s. = Not significant. p = Significance level (Germany, 2022–2023).

Finally, the extent to which prevention knowledge, practice and options for action in outdoor sports correlate in the coaching staff was investigated (Figure 4). It was shown that the coaches’ heat-specific knowledge does not appear to be reflected in their prevention behavior (*r* ^2^ = 0.005, n.s.).

## Discussion

Heat illnesses and in particular the medical emergency heat stroke are largely preventable. In organized sports, trainers are the key people for preventing such events. This applies to competitive sports as well as to amateur sports and to training with adults as well as with children and young people.

The C^3^O study presented here shows clear deficits among German coaches in recognizing possible symptoms of heat illnesses. Our large, representative sample also enables the identification of sport-specific deficits: Unsurprisingly, coaches in skiing achieved the worst scores, as such phenomena are of marginal importance in alpine environments. Soccer coaches, on the other hand, were the second least knowledgeable.

With a mean score of 62 points and a maximum of 100 points, the measures currently implemented in training to protect against heat-related illnesses also showed implementation deficits and clear differences between the sports. From the coaches’ point of view, improvements in tennis, swimming and soccer are particularly easy to achieve.

The final correlation analysis showed that pure education and information campaigns would not be sufficient for future adaptation strategies in sport: There was no correlation between theoretical knowledge of typical heat-related symptoms and the practical prevention of heat-related illnesses.

### Limitations and Strengths of the Study

The weaknesses of this study include possible selection effects, fundamental objections to the official recommendations and limitations in transferability to other regions: Firstly, it cannot be ruled out that those trainers in particular were willing to participate who considered the topic to be more relevant and therefore also had more knowledge and a more mindful training practice. Secondly, isolated recommendations of the German Society for Sports Medicine and Prevention regarding heat protection should be critically examined. For example, one recommendation is to wear headgear at least during competition breaks to avoid sunstroke [20]. As long as athletes spend training and competition breaks in sufficient shade, we believe that headgear could be dispensed with so as not to hinder heat dissipation via the head during this time. A third limitation concerns the transferability of our results to other regions of the world. Comparable trainer surveys originate exclusively from the United States. In Germany, on average only 2 h of sport are taught per week in school. In addition, 49% of all German children and young people play sport in one of the 16 state sports associations in their free time [13]. Most club coaches work on a voluntary basis in a local club and usually only have a coaching license acquired on a part-time basis. For this reason, German coaches (especially in popular sports) are on average less qualified than, for example, in the United States, where sport takes place primarily in schools and usually under the direction of professionally trained coaches. These historical and political differences mean that our collective is significantly older and is active as a trainer for longer than the coaching collectives in the United States [7, 19, 23].

The strengths of our study include the large sample size, its representativeness and the ability to differentiate between the most relevant sports in this context.

### Relevance in the International Context

The prevention of heat-related illnesses in sport is particularly advanced in the United States [23, 24]. For example, the National Athletic Trainers’ Association (NATA) has published a position statement on the prevention and treatment of external heat illnesses [25]. Their implementation has already been intensively evaluated in coaching associations [26], colleges [7, 12], middle schools [19] and high schools [12, 19, 27–29]. The clear focus was on the two most important measures for recognizing and treating the particularly dangerous heat stroke, namely, the measurement of rectal temperature, which is evidence-based on the one hand and practiced very differently among trainers on the other, and the cold-water immersion as an aggressive cooling measure, which is also controversially discussed among trainers [7, 23, 26, 29]. In addition, a task force in the USA has developed guidelines for preseason heat acclimatization for all secondary schools [30]. This includes precisely developed training plans and recommendations on clothing, such as not wearing helmets and shoulder protectors in American football during parts of the training.

In comparison, the preventive measures against heat stress published by the German Society for Sports Medicine and Prevention have a much broader approach (and have never been evaluated to date). They do not focus on diagnosis and intervention in the case of heat stroke as the heat-related illness with the highest mortality rate. Rather, they are primarily preventive, low-threshold and based on international recommendations such as postponing training [24, 31], reducing the training load [11, 24, 31], internal pre-, per- and post-cooling through specific guidelines on weight-adapted fluid intake [4, 24, 31, 32], the wearing of headgear and light, airy clothing [11, 31] as well as the use of external cooling methods [31, 32], the monitoring of heat symptoms [4, 11, 24, 31] and the presence of medical personnel during competitions [31, 32]. Most of the above-mentioned measures require little preparation in terms of time and monetary resources. Encouraging athletes to drink before, during and after training should be a training routine - not just in hot weather. Even the provision of external cooling methods can be realized with a water bucket with towels, spray bottles or reusable cool packs with little financial and time expenditure. It is therefore all the more surprising that even these simple measures are apparently not implemented in every training session and in every affected sport.

### Conclusion

Our findings suggest that newly imparted knowledge (e.g., in coach training) should be flanked by structural measures on the part of the associations and clubs in order to support coaches in converting their training. There are elaborate and differentiated concepts for this in sport [31, 32]. Structural measures for heat protection include technical and constructional measures (e.g., artificial shading, installation of water dispensers) [31–33], organizational measures (e.g., rule changes, additional shade and drinking breaks, more frequent player changes, redesign of endurance competitions as night runs) [6, 31–33] and personal measures (e.g., provision of cooling vests) [31].

Studies from the USA show that heat-specific health risks are assessed very differently within the coaching community [19, 29]. In qualitative studies, some coaches expressed an attitude that no emergency had occurred to date and that there was therefore no need for precautions [34]. Such an opinion is highly sensitive, as coaches are role models and authority figures who, among other things, decide on starting places and nominations. Marshall [11] also pointed out that such an attitude on the part of the coach is particularly fatal for young athletes, as they often do not have the courage to interrupt their training - for example, during structured exercise drills - to take breaks for drinking or shade.

With regard to climate models, current forecasts assume that in around 60 years’ time, the Summer Olympics will only be able to be held in 33 cities worldwide under medically acceptable temperature risks. At the beginning of the 22nd century, there will only be four suitable cities on our planet. Considering that such forecasts relate to health risks for highly selected and more heat-resistant competitive athletes, the question arises: is the much larger amateur sports sector sufficiently prepared for future heat extremes [35]. According to the C^3^O study, one possible starting point for improvements appears to be trainers as role models, authority figures, multipliers and managers.
