# Supplementary material for: Gaps in Heat-Related Knowledge, Practices and Adaptation Strategies Among Coaches in German Outdoor Sports
Source: Int J Public Health. 2024 Dec 4;69:1607928. doi: 10.3389/ijph.2024.1607928 (PMC11653017; doi:10.3389/ijph.2024.1607928)
Supplement: Supplementary file 1 [file Table1.docx]

| **Federal state** | **Soccer** | **Tennis** | **Mountain**  **sports** | **Athletics** | **Equestrian sports** | **Golf** | **Swimming** | **Skiing** | **Sailing** | **Cycling** | **Total n** | **Sample shares by federal state (%)** | **Population by federal state at 31.12.2022 (%)** |
| --- | --- | --- | --- | --- | --- | --- | --- | --- | --- | --- | --- | --- | --- |
| **North Rhine-Westphalia** | 26 | 26 | 26 | 26 | 26 | 26 | 26 | 26 | 26 | 26 | 260 | 22 | 22 |
| **Bavaria** | 19 | 19 | 19 | 19 | 19 | 19 | 19 | 19 | 19 | 19 | 190 | 16 | 16 |
| **Baden-Württemberg** | 16 | 16 | 16 | 16 | 16 | 16 | 16 | 16 | 16 | 16 | 160 | 13 | 13 |
| **Lower Saxony** | 12 | 12 | 12 | 12 | 12 | 12 | 12 | 12 | 12 | 12 | 120 | 10 | 10 |
| **Hesse** | 9 | 9 | 9 | 9 | 9 | 9 | 9 | 9 | 9 | 9 | 90 | 8 | 8 |
| **Rhineland-Palatinate** | 6 | 6 | 6 | 6 | 6 | 6 | 6 | 6 | 6 | 6 | 60 | 5 | 5 |
| **Saxony** | 6 | 6 | 6 | 6 | 6 | 6 | 6 | 6 | 6 | 6 | 60 | 5 | 5 |
| **Berlin** | 5 | 5 | 5 | 5 | 5 | 5 | 5 | 5 | 5 | 5 | 50 | 4 | 4 |
| **Schleswig-Holstein** | 4 | 4 | 4 | 4 | 4 | 4 | 4 | 4 | 4 | 4 | 40 | 4 | 4 |
| **Brandenburg** | 4 | 4 | 4 | 4 | 4 | 4 | 4 | 4 | 4 | 4 | 40 | 4 | 4 |
| **Saxony-Anhalt** | 3 | 3 | 3 | 3 | 3 | 3 | 3 | 3 | 3 | 3 | 30 | 3 | 3 |
| **Thuringia** | 3 | 3 | 3 | 3 | 3 | 3 | 3 | 3 | 3 | 3 | 30 | 3 | 3 |
| **Hamburg** | 3 | 3 | 3 | 3 | 3 | 3 | 3 | 3 | 3 | 3 | 30 | 3 | 3 |
| **Mecklenburg-Western Pomerania** | 2 | 2 | 2 | 2 | 2 | 2 | 2 | 2 | 2 | 2 | 20 | 2 | 2 |
| **Saarland** | 1 | 1 | 1 | 1 | 1 | 1 | 1 | 1 | 1 | 1 | 10 | 1 | 1 |
| **Bremen** | 1 | 1 | 1 | 1 | 1 | 1 | 1 | 1 | 1 | 1 | 10 | 1 | 1 |
| **Total n** | 120 | 120 | 120 | 120 | 120 | 120 | 120 | 120 | 120 | 120 | 1,200 | 100 | 100 |

Supplement: Distribution of the C^3^O sample by federal state and type of sport as well as sample and population shares by federal state (n_weighted_)
